# Chemical Properties and Biological Activity of Bee Pollen

**DOI:** 10.3390/molecules28237768

**Published:** 2023-11-25

**Authors:** Cristina Rodríguez-Pólit, Rebeca Gonzalez-Pastor, Jorge Heredia-Moya, Saskya E. Carrera-Pacheco, Fabián Castillo-Solis, Roberto Vallejo-Imbaquingo, Carlos Barba-Ostria, Linda P. Guamán

**Affiliations:** 1 Centro de Investigación Biomédica (CENBIO), Facultad de Ciencias de la Salud Eugenio Espejo, Universidad UTE, Quito 170527, Ecuador; rebeca.gonzalez@ute.edu.ec (R.G.-P.); jorgeh.heredia@ute.edu.ec (J.H.-M.); saskya.carrera@ute.edu.ec (S.E.C.-P.); fabianc.castillo@ute.edu.ec (F.C.-S.); 2 Centro de Referencia Nacional de Genómica, Secuenciación y Bioinformática, Instituto Nacional de Investigación en Salud Pública “Leopoldo Izquieta Pérez”, Quito 170403, Ecuador; crodriguez@inspi.gob.ec; 3 Escuela de Salud Pública, Universidad San Francisco de Quito USFQ, Quito 170527, Ecuador; 4 Departamento de Estudios Organizacionales y Desarrollo Humano DESODEH, Facultad de Ciencias Administrativas, Escuela Politécnica Nacional, Quito 170525, Ecuador; roberto.vallejo@epn.edu.ec; 5 Escuela de Medicina, Colegio de Ciencias de la Salud Quito, Universidad San Francisco de Quito USFQ, Quito 170901, Ecuador; cbarbao@usfq.edu.ec; 6 Instituto de Microbiología, Universidad San Francisco de Quito USFQ, Quito 170901, Ecuador

**Keywords:** natural product, pollen, antimicrobial, bioactivity, biological activity

## Abstract

Pollen, a remarkably versatile natural compound collected by bees for its abundant source of proteins and nutrients, represents a rich reservoir of diverse bioactive compounds with noteworthy chemical and therapeutic potential. Its extensive biological effects have been known and exploited since ancient times. Today, there is an increased interest in finding natural compounds against oxidative stress, a factor that contributes to various diseases. Recent research has unraveled a multitude of biological activities associated with bee pollen, ranging from antioxidant, anti-inflammatory, antimicrobial, and antifungal properties to potential antiviral and anticancer applications. Comprehending the extensive repertoire of biological properties across various pollen sources remains challenging. By investigating a spectrum of pollen types and their chemical composition, this review produces an updated analysis of the bioactive constituents and the therapeutic prospects they offer. This review emphasizes the necessity for further exploration and standardization of diverse pollen sources and bioactive compounds that could contribute to the development of innovative therapies.

## 1. Introduction

Pollination has a paramount impact on agriculture and it is directly tied to food security. A large number of edible crops worldwide, including all food and industrial plant-based products, require pollination by bees [1]. In other words, bees carrying pollen from plant to plant are responsible for a third of the world’s food production, according to specialists at the United Nations’ Food and Agriculture Organization (FAO). Pollinated plants provide more than half of the world’s fats and oils for our diets [2]. Many experts ensure that the planet’s ecosystem would not survive without pollinators and bee pollen [3].

Pollen, the male gametophyte of blooming plants, is a source of high-energy food stored as a food reserve by bees and other insects [4]. For instance, bee pollen comes from plant pollen gathered by bees and mixed with nectar or the insects’ salivary gland secretion. In this state, it is carried to hives. There, bees combine it with their saliva and pack it into honeycombs, which are then covered in a wax–honey combination. Anaerobic fermentation continues under these conditions, creating lactic acid as a preservative. The substance created in this manner serves as a source of nutrition for both adult and larval bees [5].

Since ancient times, pollen and bee products have been among the most popular natural products, used both for their nutritional benefits and for various medical uses, given their potent therapeutic properties and many bioactive molecules [6]. Today, people use pollen as a dietary supplement, as well as for some alternative medicinal treatments. Also, pollen constitutes a concentrated, nutrient-dense food that is high in energy and vitamins. Pollen has important potential as a “supplement and survival food” and has recently been used for conditioning in athletic fitness [4]. Bee pollen, a complex mixture of plant pollens collected by bees, exhibits significant variations in its macro- and microelement composition, determined by factors such as floral origins, geographical location, soil type, and climate conditions [7]. The nutritional content and biological activities of bee pollen are directly linked to the factors mentioned above, as well as the diverse plant species from which it is collected. Also, extraction methods employed in collecting bee pollen can impact its nutrient concentration and biological activity [8]. Research indicates these factors contribute to differences in bee pollen’s antioxidant, anti-inflammatory, antimicrobial, anticarcinogenic, and nutritional properties [6]. Scientific studies credit bee products, including pollen, with numerous bioactive compounds and positive health effects, including antioxidant, antibacterial, anti-inflammatory, and anticancer capabilities (among others) [9,10].

Our review addresses a critical gap in the existing research on bee pollen by consolidating data on its diverse chemical compositions and biological activities, thereby offering a more robust and comprehensive analysis of its therapeutic potential. We focus on the specific bioactive compounds responsible for bee pollen’s observed therapeutic effects, an aspect frequently overlooked due to the general tendency to treat bee pollen as a uniform entity, despite its inherent chemical diversity.

This manuscript compiles recent findings of a wide range of biological activities associated with bee pollen and its components. It also discusses the complexities in comprehending the diversity and composition of various pollen sources. Furthermore, it provides an updated review of the bioactive constituents and therapeutic potential of different types of pollen, underscoring the necessity for additional research and standardization in this field.

## 2. Chemical Composition and Nutritional Value

For bees, the consumption of nectar represents their main intake of sugars, while pollen means a source of protein, lipids, and micronutrients, yet its content can widely vary, depending on the collection of bee and plant species [11] (Table 1). Also, it is important to consider its high content of sugars as nectar, and salivary secretions are added to agglutinate pollen into pellets for transport and storage [6,12].

Carbohydrates account for approximately two-thirds of pollen’s dry weight. Nevertheless, their contents vary significantly, depending on factors such as the plant species, growth conditions, and harvesting methods. Bee pollen contains monosaccharides (fructose and glucose) and disaccharides (sucrose, turanose, maltose, trehalose, etc.), with the fructose/glucose ratio typically ranging from 1.20 to 1.50 [12]. Additionally, bee-collected pollen exhibits higher levels of reducing sugars compared to plant pollen. Polysaccharides like sporopollenin, found in the outer layer of pollen grains (exine), provide structural integrity and protection to the pollen contents. In contrast, the inner layer (intine) consists of cellulose and pectin, which play a role in biological functions but do not contribute to nutritional value [12].

Pollen grains differ from one another in shape and in the content of bioactive and nutritional substances, due to geographic and botanical factors, climatic conditions, and the various processes they are subjected to for commercial production [12,16,17]. Compared with monofloral pollen, heterofloral bee pollen combines the biochemical and organoleptic qualities of the plants of origin and has the highest bioactive content [18]. However, since diverse extraction and analysis techniques have been used to identify the chemical characteristics of pollen, its composition also tends to vary (Table 2) [13,16]. Proteins, enzymes, essential and non-essential amino acids, carbohydrates, lipids, fatty acids, phenolic compounds, vitamins, and a small number of other compounds are among pollen’s key nutrients and bioactive substances [19]. 

The type of pollen found in a geographical location will depend on the kind of crops in the area and, as a result, some bioactive compounds will be present in more significant amounts than others. For example, the analysis of monofloral pollen from diverse plant species in Romania found that *Prunus* L. sp., *Aesculus* sp., and *Brassica* sp. are important protein sources [22]. A high concentration of the same nutrient was found in *Bombax ceiba* pollen from India [23]. *Plantago lanceolata* pollen and *Brassica* sp. pollen from Romania have high carbohydrate and lipid concentrations, respectively [22], while *Rosa rugosa* and *Gingko biloba* L. pollen from China are also high in lipids [24].

*Castanea sativa* bee pollen is an important type of pollen collected in the northwest of the Iberian Peninsula, and its phenolic components have been investigated extensively [25,26]. Pollen with a high concentration of polyphenolic compounds has been shown to exhibit a variety of biological activities; as a result, these compounds have been among the most investigated ones. For example, pollen from *Eucalyptus* spp. [27] and *Helianthus annuus* L. [28] displays a substantial inhibitory effect against tyrosinase, while pollen from *Miconia* spp. [27] has antihemolytic activity. Pollen from *Cocos nucifera*, *Miconia* spp., [27] *Mimosa diplotricha* [28], *Brassica napus* [29], coriander [30], *Olea europaea*, and *Ononis spinosa* [31] has also been shown to have antioxidant activity. Furthermore, anticancer activity from *Olea europaea* pollen [31] and antiparasitic activity from *Attalea funifera* pollen [32] have been observed as a result of these compounds.

Pollen can be considered a high-value nutritional supplement for the human diet, particularly, as it contains essential amino acids that play an important role in functions such as nutrient absorption and gene expression, among others [15]. The protein amount found in bees’ pollen hinges on the plant type, usually falling between 15% and 40% of dry weight. For instance, the protein amount in bee-gathered pollen from eucalyptus species is about 24.9 ± 0.6% of dry weight. However, the protein content is lower in sunflower pollen, registering just 13–15% post bee collection [11]. The main amino acids in bee pollen include leucine, lysine, valine, aspartic acid, glutamic acid, and proline [33]. These amino acids are relevant for the proper functioning of the body, and their presence in bee pollen makes it a highly nutritious food [34].

The nutritional composition of bee pollen includes carbohydrates, proteins, lipids, vitamins, minerals, polyphenols, and a relatively small percentage of other constituents. Due to variation on its chemical composition, it is challenging to establish a fixed dietary value of bee pollen. While pollen holds potential as a source of macronutrients for human consumption, reliance on it to fully meet dietary requirements remains improbable. To illustrate, a 100 g portion of eucalyptus pollen, approximating a protein quantity of 24.9 g, would deliver nearly 53.9% of the estimated average requirement (EAR) for an adult weighing 70 kg, assuming it is entirely assimilable. Nevertheless, regular intake of such a volume of pollen would be atypical—being considered, for instance, as a supplementary food [4].

Furthermore, the assimilation of nutrients is compromised by the robust outer layer(exine) of the pollen grain, restricting the utilization of its nutrient reserves. To enhance the nutritional potential of bee pollen, a myriad of methods has been explored. These encompass chemical and mechanical treatments, physical treatments like ultrasound, supercritical fluids, and biotechnological approaches such as fermentation and enzymatic hydrolysis [15]. Of these, enzymatic hydrolysis, pioneered using proteases, has emerged as the most fruitful, amplifying the nutritional content of proteins by 13–18%, phenols by 83–86%, and flavonoids by 85–96% [15].

Regarding the characterization of the nutrient content of pollen, researchers face certain challenges, as outlined in previous studies [4]. The application of varied analytical techniques may result in dissimilar measurements for the same type of nutrient. Table 2 summarizes some nutrient content-measuring mechanisms.

Such variances can depend on an array of factors, such as the efficiency of the extraction process or interference from other substances present in pollen. Furthermore, variations in nutrient content can be attributed to pollen collection and preparation methods, including factors such as bees incorporating nectar or honey, the specific techniques employed for drying and storage, and any anomalies that may impact nutrients, such as contamination or degradation. Adding to the complexity is the unique structure of pollen grains, featuring a tri-layered exterior wall that can affect solvent permeability and subsequent nutrient availability. To enhance precision and consistency in results, it is recommended to disrupt the pollen wall before conducting analyses [8].

**Figure 1:**
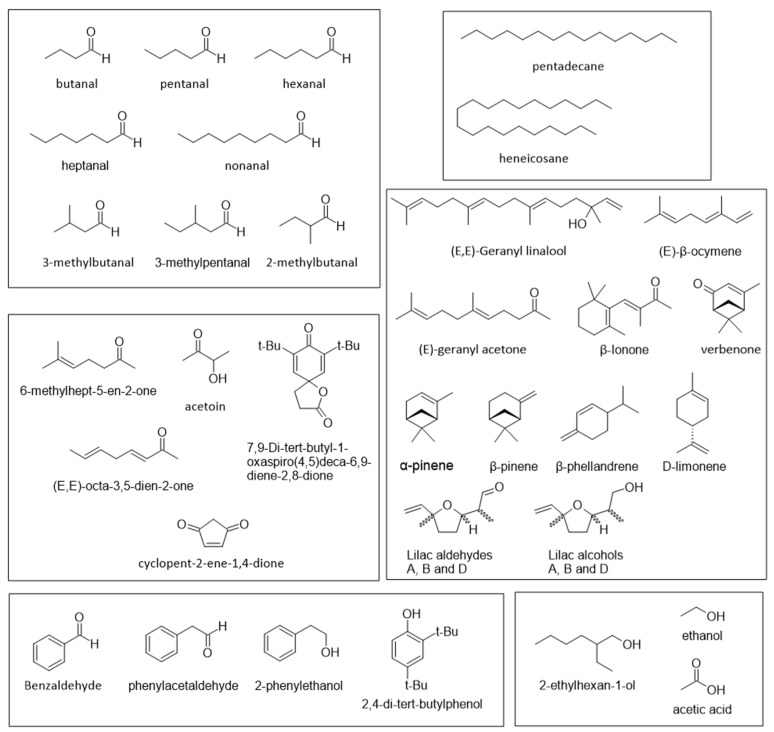
Volatile compounds found in bee pollen.

The analysis of the volatile compounds found in pollen revealed the existence of numerous types of molecules, the most abundant of which were aldehydes (butanal, pentanal, hexanal, heptanal, 3-methylbutanal, etc.), saturated and unsaturated, linear and branched hydrocarbons (pentadecane, heneicosane, etc.), ketones (6-methylhept-5-en-2-one, acetoin, (*E*,*E*)-octa-3,5-dien-2-one, etc.), alcohols (2-ethylhexan-1-ol, ethanol, etc.), benzene derivatives (benzaldehyde, 2-phenylethanol, phenylacetaldehyde, etc.), and different terpenoids (α-pinene, (*E*,*E*)-geranyl linalool, (*E*)-β-ocymene, Lilac aldehydes, etc.) [34,35,36]. Figure 1 shows a few examples of these compounds.

Although some short-chain acids, such as acetic acid, butanoic acid, and hexanoic acid, have been found [34], the majority of carboxylic acids and their derivatives are found in the lipid fraction and correspond to fatty acids, both saturated and unsaturated, with unsaturated fatty acids being the most abundant [24,37], with a total unsaturated to saturated fatty acid ratio ranging from 2.2 to 6.7 [38].

Humans require lipids, various essential fatty acids, and antioxidants for growth, healthy development, and protection against diseases. In addition, fatty acids are a critical component of membrane phospholipids [12]. Nevertheless, the proportion of lipid content in pollen also may differ based on the type of plant. For instance, eucalyptus pollen gathered by bees has a meager lipid content of 0.6–1.9% dry mass, while canola pollen has an exceptional 32% dry weight; also, bee-collected almond pollen contains components like fatty acids, sterols, vitamins, and minerals [11].

Palmitic acid, stearic acid, and capric acid are among the most common fatty acids [35,39] Unsaturated fatty acids include α-linolenic acid (ALA), linoleic acid (LA), arachidonic acid (ARA), tetracosenoic acid, oleic acid, erucic acid, *cis*-11-eicosenoic acid, *cis*-4,7,10,13,16,19-docosahexanoic acid, eicosatrienoic acid, eicosapentaenoic acid, heptadecenoic acid, petroselinic acid, and dihomo-γ-linolenic acid [24,38,39,40] Because of the high content of unsaturated acids in various pollen samples, pollen is recognized as an essential source of omega-3 fatty acids in the human diet [38]. Carotenoids and phytosterols (particularly β-sitosterol) have also been found in this fraction [15].

**Figure 2:**
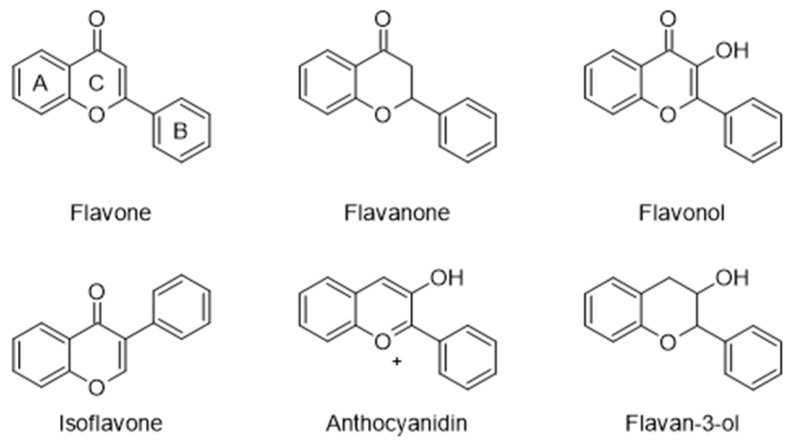
Structure of the flavonoids.

Some of the bioactive constituents found in pollen are phenolic compounds [30], produced by plants as secondary metabolites, with flavonoids as the most significant polyphenolic class. Flavonoid glycosides and free aglycones are the most common flavonoid forms in bee pollen, which are richer in ethanolic extracts than aqueous extracts. However, water, followed by ethanol and methanol, produced the highest concentration of bee pollen flavonoids and terpenoids, whereas less polar flavonoids are extracted using nonpolar solvents [41]. The general structure of flavonoids is a 15-carbon skeleton with two phenyl rings, A and B, connected by a pyran ring, C (Figure 2). Flavones, flavanones, flavonols, isoflavones, anthocyanidins, and flavan-3-ol are the six subclasses of flavonoids [42].

**Figure 3:**
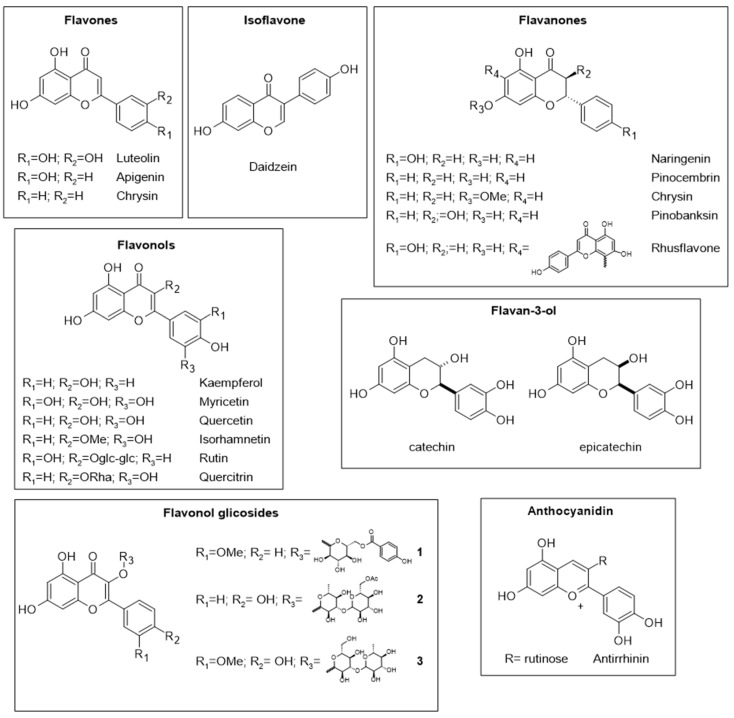
Some flavonoids found in bee pollen.

Several major flavonoids have been found in various pollen samples (Figure 3), including luteolin, apigenin, chrysin (flavones), naringenin, pinocembrin, pinostrobin, pinobanksin (flavanones), rutin, myricetin, quercetin, kaempferol, isorhamnetin, quercitrin (flavonols), daidzein (isoflavone), antirrhinin (anthocyanidin), catechin, and epicatechin (flavanols) [30,35,39,40,43,44]. Other flavonoids identified recently include the biflavonoid rhusflavone, which exhibits high leishmanicidal activity [32], and several flavonoid glycoside derivatives such as the monobenzoylated pyranose **1**, the mono-acetylated kaempferol diglycoside **2**, and isorhamnetin-*O*-hexosyl-deoxyhexoside **3** [25,45].

**Figure 4:**
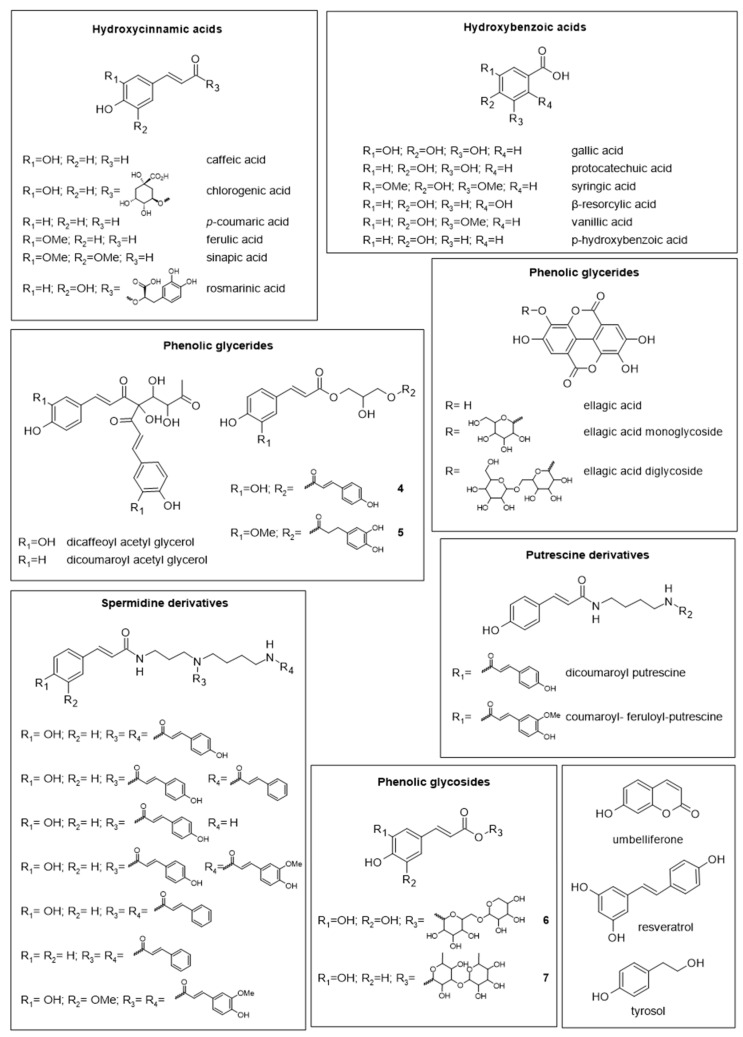
Some phenolic compounds found in bee pollen.

Phenolic acids and their derivatives are other phenolic compounds found in pollen (Figure 4). Polar phenolic acids may be extracted using a variety of water–solvent solutions, including ethanol–water and methanol–water [41]. Caffeic, chlorogenic, *p*-coumaric, ferulic, sinapic, gallic, protocatechuic, syringic, β-resorcylic, vanillic, *p*-hydroksybenzoic, and rosmarinic acid are among the main phenolic acids reported [32,35,39,40,43,44,46]. Furthermore, cinnamic acid amide derivatives, including spermidine [25,28,30,47,48,49] and putrescine derivatives [32,50,51], and phenolic glycerides, like 1,3-*O*-coumaroyl-caffeoyl-glycerol (**3**) and 1,3-*O*-feruloyl-dihydrocaffeoyl glycerol (**4**), have also been identified [16], along with phenolic glycosides like 3,4,5-trihydroxycinnamic acid diglycoside (**5**) and 3-hydroxychinamic acid dirhamnoside (**6**) [45]. The compounds mentioned above are cinnamic or benzoic acid derivatives; however, polyphenols with other structures have been identified, including tyrosol [40] umbelliferone [43], ellagic acid [43], various glycosylated derivatives [45], and phytoalexines like resveratrol [40,44].

**Figure 5:**
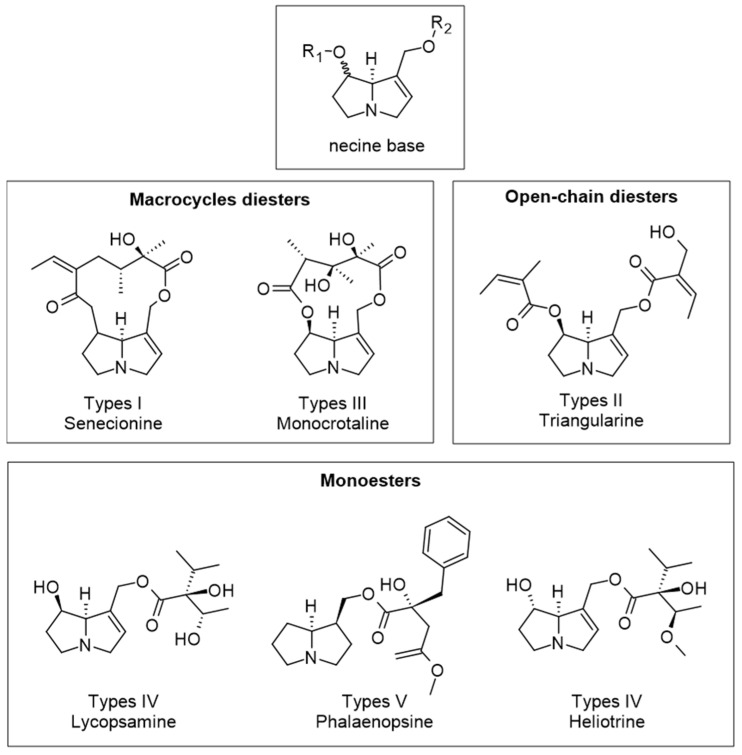
Examples of pyrrolizidine alkaloids showing the five main types.

Pyrrolizidine alkaloids, secondary plant defense compounds, have also been identified in significant amounts in bee pollen [52]. These harmful natural pollutants are produced by plants in the summer and their structures vary, depending on the origin of the investigated pollen; nonetheless, these types of molecules share a structural component identified as a 1-azabicyclo[3.3.0]octane backbone, which also contains an additional 1,2-double bond, a hydroxymethyl substituent in the 1-position, and a hydroxyl group in the 7-position. The resulting pyrrolizidine alkaloid core, known as the necine base, can be mono- or diesterified with acyl moieties with different structural and stereochemical features. These alkaloids are categorized into five groups, based on the type of acyl groups and the degree of esterification present, and examples of these types are shown in Figure 5 [52].

**Figure 6:**
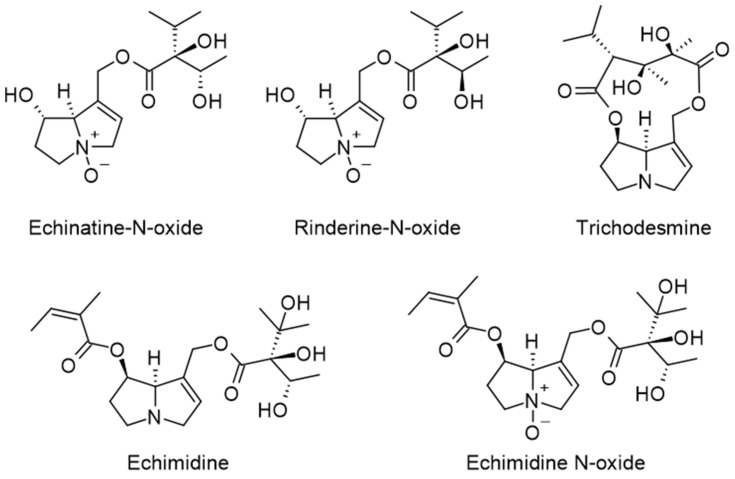
Pyrrolizidine alkaloids found in bee pollen.

The concentration and types of alkaloids observed vary significantly, due to pollen origin diversity, with tertiary heterocyclic amines and their related N-oxide form being the two main forms of these alkaloids. It has been found in several analyzed samples, for example, the presence of echinatine N-oxides and rinderine N-oxide [52], trichodesmine [53], or echimidine and echimidin N-oxid [54] (Figure 6).

## 3. Anti-Inflammatory Activity

Inflammation is a complex biological process that, through immune and non-immune cells, aims to protect the body from pathogenic agents. This process also promotes the repair of damaged tissues caused by infections, injuries, or damage caused by exposure to toxic agents, etc. [55,56] However, persistent inflammation can lead to disruptions in metabolism and the initiation of numerous long-term conditions, including sarcopenia, metabolic syndrome, and neurodegenerative disorders [57,58]. Also, depending on the severity of the causal agent, this response can be acute or chronic (extensive), triggering diseases that account for 50% of deaths related to chronic inflammation, such as renal failure, diabetes mellitus, ischemic heart disease, and cancer [59]. Considering these complications, the most prevalent treatment methods for these conditions involve a category of widely used medications known as “anti-inflammatories” [60].

However, several studies point out that the prolonged intake of these drugs may cause cardiovascular, hepatic, gastrointestinal, pulmonary, renal, and cerebral complications [60]. In this context, the pursuit of substances possessing anti-inflammatory characteristics and minimal negative impacts on health holds significant importance. The main viable options involve substances of natural origin, as they have demonstrated effective biocompatible therapeutic properties that are also cost-effective in combating inflammation [61,62]. One of the most relevant examples is “bee pollen,” a natural product that combines molecules of plant origin with substances present in bee fluids, generating a complex rich in proteins, carbohydrates, lipids, dietary fibers, minerals, and secondary metabolites such as carotenoids, flavonoids, and vitamins (A, C, and E) [19,62].

A study evaluated the anti-inflammatory activity of three bee-pollen extracts obtained from *Camellia sinensis* (BP-Cs), *Brassica campestris* (BP-Bs), and *Nelumbo nucifera* (BP-Nn). As a starting point, an inflammation model was designed by adding lipopolysaccharides (LPS) to raw 264.7 cells (mouse macrophages) [63]. The extract that showed the highest anti-inflammatory activity was BP-Cs, as its high polyphenol content reduced the expression of genes associated with inflammation and blocked the NF-kB and MAPK signaling pathways. These results were followed up with an in vivo study in a mouse model with acute lung injury. Metabolomic analysis of mouse serum corroborated its capacity to reduce inflammation [63]. In a similar study, the anti-inflammatory activity of extracts from eight samples of heterofloral bee pollen obtained from northeast Algeria were evaluated. This study was conducted in vivo using the formalin-induced paw edema method in rats, showing that most of the evaluated extracts presented anti-inflammatory activity. The extract with the highest concentration of flavonols showed better activity than the reference anti-inflammatory drug they used (diclofenac, 20 mg/kg of body weight) [64].

In another study, it was evidenced that bee pollen exhibited a better anti-inflammatory advantage compared to other bee-derived products; this was due to the administration of 300 mg/kg/day of bee pollen for 7 days in an inflammation model in Dawley rats in which edema was induced, increasing anti-inflammatory cytokines such as IL-4, IL-10, and IL-1RA, and a reduction in pro-inflammatory cytokines such as IL-1β, IL-6, and TNF-α [65]. Another study evaluated the anti-inflammatory activity of stingless bee pollen from *Melipona fasciculata* obtained from three places in the state of Maranhão, Brazil [66]. Under in vitro conditions, the bee pollen extract from Chapadinha (EHPC) showed the best ability to inhibit cyclooxygenases (COX), key enzymes in the inflammatory process [67].

At 10 µg/mL, EHPC inhibited 100% of COX-2 and 27% of COX-1. This led to an in vivo study in two inflammation assays induced by carrageenan and dextran. The first assay demonstrated that 250 mg/kg reduced 100% of carrageenan-induced inflammation compared to the saline control, and its effect was comparable to the anti-inflammatory drug indomethacin. In the second assay, it was determined that after 5 h, 500 mg/kg of EHPC could inhibit between 66–100% of dextran-induced inflammation, showing higher anti-inflammatory activity than the drug ciproheptadine [66].

Also, the methanolic extract of bee pollen was evaluated in a Wistar rat inflammation model, where sterile air-induced pouches were followed by carrageenan-induced inflammation. The results demonstrated that administering 200 mg of bee pollen extract in each pouch reduced exudate volume by 40%, compared to the carrageenan control. Moreover, administering 100 and 200 mg of extract reduced the number of leukocytes in the exudate by 63% and 74%, respectively, and decreased the granulation tissue weight by 34% and 30.2%, respectively [60]. Apart from the ability of bee pollen polyphenols to block NF-κB and MAPK signaling pathways and reduce the gene expression related to inflammation [63], pollen has shown the ability to inhibit the activity of lipoxygenases and cyclooxygenases by interacting with amino acid residues, which prevents the conversion of arachidonic acid into toxic molecules such as prostaglandins and leukotrienes, precursors of acute and chronic inflammatory states [66,68].

Additionally, the linolenic and linoleic fatty acids present in bee pollen can bind to the histamine H1 receptor, and flavonoids can also reduce nitric oxide production, synergistically decreasing the inflammatory response [69]. However, bee pollen proves to be an excellent alternative compared to other anti-inflammatory drugs [68,69,70]. Its wide range of constituents and variability need further studies with greater rigor in the chemical and clinical fields [69]. Some research indicates that it may cause adverse effects in individuals prone to allergic reactions to pollen [71]. This suggests the need to keep searching for alternatives to optimally exploit its constituents without causing undesirable effects by standardizing efficient extraction and fermentation methods.

## 4. Antioxidant Activity

One of the most crucial qualities of pollen is its antioxidant ability, which helps to prevent several diseases by shielding cells from oxidative agents like free radical damage. Antioxidants are chemicals that can block or slow down the oxidation of other molecules, stopping alterations and mutations that can lead to several health conditions. Plant antioxidants that are found in honey and other bee products exhibit high bioactivity and molecular diversity [6]. Interestingly, a comparison of the antioxidant activity from organic pollen with values from honey suggested a higher antioxidant activity of organic pollen than that of honey [72].

Most bee pollen’s biological activities have been linked to its antioxidant properties. These characteristics are attributed to their high polyphenolic content and strong ability to eliminate free radicals [73]. For instance, polyphenols, such as flavonoids found in bee pollen, can inactivate reactive oxygen species (ROS), electrophiles, scavenging free radicals, preventing them from becoming mutagens [1,62]. The antioxidant capacity of bee pollen samples often positively correlates to their polyphenolic concentration. However, some studies also suggest that antioxidant activity in bee pollen extracts is not always connected with large levels of phenolic compounds, implying that the antioxidant activity is not confined to phenolic chemicals [73]. Other molecules present in bee pollen, such as carotenoids, glutathione, phytoalexins, and vitamins C and E, have also been found to contribute to its antioxidant properties [1,74]. Thus, the antioxidant capacity of bee pollen is highly dependent on its particular composition, which can vary between samples depending on their origin [5,74] An extensive list of bee pollen antioxidant properties based on their composition and origin was presented by Tutun et al. in 2021 [44].

**Figure 7:**
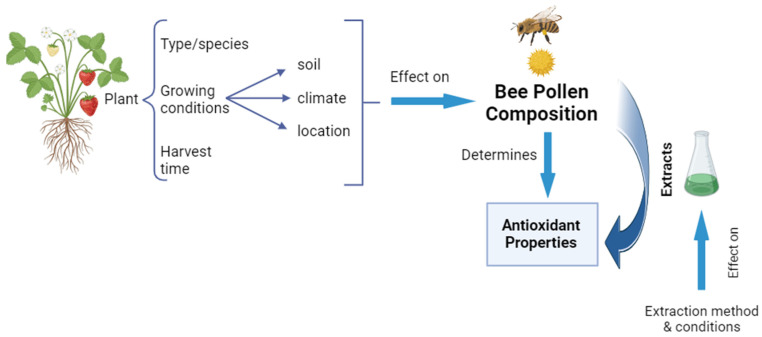
Aspects influencing bee pollen antioxidant properties. Figure created with BioRender.com (accessed on 17 October 2023).

As shown in multiple studies, there are significant differences in antioxidant activity, chemical component concentration, and types among pollen grains from different plant species and geographical locations [74,75,76]. The type of plant from which bee pollen originates, the growing conditions of the plants, such as soil or climate, and the time of harvest, affect both the content and the characteristics of the pollen [5]. Various direct and indirect in vitro techniques, such as Trolox equivalent antioxidant capacity (TEAC), oxygen radical absorbance capacity (ORAC), DPPH, ABTS+, FRAP, and the b-carotene bleaching method [77], have been used to evaluate the antioxidant capabilities of bee pollen. Usually, these assays are carried out using extracts instead of the raw material, due to the increased amounts of bioactive compounds. However, the composition of the extracts is influenced by the extraction method and conditions used [5]. In this regard, Kim et al. [78] described an optimized method for bee pollen extraction, which can benefit the analysis of the antioxidant activity of different samples. Overall, it is necessary to analyze each batch of pollen to elucidate its particular composition and specific antioxidant potential. Figure 7 illustrates some of the main aspects affecting bee pollen composition and, therefore, its antioxidant properties.

## 5. Antimicrobial Activity

There is a growing consumer interest in natural health products and alternative remedies. Natural products, such as bee pollen, often contain a complex mixture of compounds that can act synergistically, enhancing their antimicrobial effects. The bioactive compounds present in bee pollen, such as glucose oxidase, plant phenolics, flavonoids, and secondary metabolites, contribute to its antimicrobial properties [79]. These findings highlight the potential of bee pollen as a natural antimicrobial agent with applications in various fields, including food and medicine [80].

### 5.1. Viruses

Bee pollen has been found to have the potential to inhibit certain viral replications. Several studies have investigated the therapeutic properties of bee pollen and its effects on viral infections. One study, by Komosińska-Vassev et al. (2015), discussed the chemical composition of bee pollen, which includes various substances such as amino acids, lipids, vitamins, and flavonoids. These compounds may contribute to the antiviral activity of bee pollen [68]. Another study, by Denisow and Denisow-Pietrzyk (2016), highlighted the anti-inflammatory properties of bee pollen, which can be attributed to the presence of fatty acids and phytosterols. Inflammation is often associated with viral infections, and the ability of bee pollen to reduce inflammation may indirectly inhibit viral replication [1]. Furthermore, Alaux et al. (2011) assessed the effects of pollen nutrition on bees’ immune response to viral infections. They found that pollen nutrients can help bees fight against parasites and pathogens, including viruses. This suggests that bee pollen may similarly inhibit viral replication in other organisms [81]. Furthermore, Corona et al. (2023) conducted experiments on honey bees and found that competition for nutritional resources, including pollen, can reduce viral replication. They observed that pollen feeding increased viral replication in varroa-parasitized bees, indicating a potential relationship between pollen consumption and viral inhibition [82]. In addition, a study by Didaras et al. (2022) evaluated the antiviral activity of Greek bee bread and bee-collected pollen against enterovirus D68. The results showed that bee bread and bee-collected pollen exhibited potent antiviral activity against the virus. This suggests that bee pollen may have broad-spectrum antiviral effects [83]. Overall, these studies provide evidence of bee pollen’s potential to inhibit certain viral replications. The chemical composition of bee pollen, its anti-inflammatory properties, and its ability to enhance immune responses may contribute to its antiviral activity. Further research is needed to understand the mechanisms underlying this potential fully and to explore the use of bee pollen as a natural antiviral agent.

### 5.2. Bacteria

Several studies have demonstrated the antibacterial activity of different pollen extracts against various pathogenic bacteria. For example, a study by Fatrcová-Šramková et al. assessed the antibacterial activity of monofloral bee pollen against pathogenic bacteria and found that *Staphylococcus aureus* was the most sensitive bacteria to an ethanol extract of poppy pollen, while *Salmonella enterica* was the most sensitive bacteria to a methanol extract of rape bee pollen and an ethanol extract of sunflower pollen [1]. Similarly, the antibacterial properties of pollen extracts from different plant species found that bee-pollen extracts obtained from plants in the family *Papaveraceae*, *Brassicaceae*, and *Asteraceae* could inhibit the growth of *Bacillus subtilis*, *Escherichia coli*, *Klebsiella* spp., *Listeria monocytogenes*, *Pseudomonas aeruginosa*, and *Staphylococcus aureus* [84]. The antimicrobial activity of pollen can be attributed to the presence of bioactive compounds such as flavonoids, phenolic compounds, and other phytochemicals [85]. Additionally, certain strains of pollen-associated bacteria, such as *Streptomyces*, have been found to produce antibiotics that inhibit honey bee pathogens [86]. It is important to note that the antimicrobial activity of pollen is selective and does not harm beneficial bacteria. Bee pollen, for example, has been found to inhibit pathogenic bacterial strains, while not affecting lactic acid starter cultures, which are beneficial bacteria [87]. This selective antimicrobial activity makes pollen a potential candidate for antimicrobial applications that do not disrupt the natural microbiota.

A recent study highlighted that pollen samples from *Punica granatum* and *Quercus ilex* pollen extracts demonstrated antibacterial activity against *Pseudomonas aeruginosa*, a bacterium notoriously resistant to a wide range of synthetic and natural antibacterial agents; this study drew a positive correlation between the antioxidant content of pollen extracts and their antibacterial capacity [88].

Furthermore, Gercek and coworkers evaluated bee-collected pollen extract (BCPE) against various food-borne pathogenic bacteria. Their results showed that MIC values against Gram-positive microorganisms ranged from 2.5 to 5 mg/mL (for *Staphylococcus aureus* NCTC 10788, MIC value 5 mg/mL; 2.5 mg/mL for other Gram-positive bacteria). MIC values for Gram-negative bacteria were between 5 and 10 mg/mL (MIC value of *Salmonella Typhimurium* RSSK 95091 5 mg/mL; for other Gram-negative bacteria, 10 mg/mL) [39].

The antimicrobial activity of bee pollen was also assessed on Clostridia organisms, i.e., *Clostridium butyricum*, *Clostridium hystoliticum*, *Clostridium intestinale*, *Clostridium perfringens*, and *Clostridium ramosum*. The results showed an antibacterial activity against all of the above-mentioned strains of clostridia [89]. Bee pollen has also been shown to be effective against some virulence factors from pathogenic bacteria. *Salmonella* Enteritidis adherence to epithelial cells was diminished from 25.6 ± 6.5 (control) to 6.7 ± 3.3 bacteria/epithelial cells in vitro when pollen was applied at dilutions 1:8 [90]. Recent studies have shown synergistic effects when bee pollen is combined with known antimicrobials. For instance, combining bee pollen and certain antibiotics amplified the antimicrobial effects against resistant bacterial strains [91].

### 5.3. Fungi

The antifungal properties of bee pollen have been investigated against a wide range of pathogenic fungi, including several species of *Candida* (*C. albicans*, *C. glabrata* and *C. krusei*) [84,92], *Aspergillus* species [93], and other pathogenic fungi [89]. However, limited research has focused on elucidating the mechanisms by which it exerts its inhibitory effects on pathogenic fungi. The antifungal activity of bee pollen is mainly attributed to its phenolic compounds and flavonoids [94]. The phenolamides, also present in bee pollen, have been reported to have promising antifungal activity [95]. It is crucial to remember that the composition of pollen derived from diverse regions, floral sources, and bee species exhibits substantial variations that pose additional challenges in understanding its antifungal potential.

As the challenge of antimicrobial resistance escalates, the importance of natural products like bee pollen becomes undeniable. Future studies should focus on the standardization of procedures for bee-pollen extraction and quantification, evaluate the safety and efficacy of bee pollen in clinical settings, and delve deeper into the molecular mechanisms underlying its antimicrobial action.

## 6. Anticarcinogenic Activity

Cancer remains the leading cause of death worldwide. Standard treatments often harm healthy cells, causing undesirable side effects; hence, research for alternative antitumor drugs has become a current focus, especially for natural products that are generally considered safer [96,97]. Several reports have identified bee products (propolis, honey, royal jelly, bee venom, bee pollen) as promising candidates to treat a variety of cancers [98,99]. In particular, bee pollen has been investigated in in vitro models of human prostate cancer, breast cancer, lung carcinoma, gastric adenocarcinoma, hepatocarcinoma, cervix carcinoma, and ovarian carcinoma. Similar to other natural products, differences in the antiproliferative activity of bee pollen have been described, depending on the extract preparation method and the bioactive composition, which are related to the region where the bee pollen was collected and the bee species [12,100,101].

Previously, a study reported that the total fraction of polysaccharides from bee pollen from *Rosa rugosa* collected from China had been observed to possess dose-dependent, synergistic, antiproliferative effects at concentrations < 5 mg/mL. In contrast, it was described that low concentrations (20 μg/mL) of crude bee-pollen extracts from four species of Indonesian stingless bees had a reduced antiproliferative activity against human cancer-derived cell lines, compared to crude extracts from propolis [100]. Later, Amalia et al. determined that the antiproliferative activity of bee pollen of *Trigona* spp. from a region of Indonesia was dose- and time-dependent, reporting an IC_50_ value of 18.6 ± 0.03 mg/mL after 24 h. More importantly, the bee pollen was less toxic to normal cells than water-soluble propolis [102]. In another report, IC_50_ values between 0.9 to >25 mg/mL were demonstrated in several human cancer-cell lines treated with bee pollen collected from different geographical origins in South Korea [103]. They also determined that heterofloral pollen grains presented better inhibitory effects than bifloral and monofloral sources. Arung et al. compared the cytotoxicity of bee pollen extracts from seven stingless bees from Indonesia and concluded that the bee-pollen extracts of *H. fimbriata* show the highest cytotoxicity profile [104]. The results seen in this study were likely related to differences described in the composition among the extracts, with varying concentrations of rutin, hydroxycinnamic acids, salicin, or ellagic acid [105,106]. Recently, strong antiproliferative effects were observed by enzymatically cleaved bee-pollen proteins (hydrolysates) at an IC_50_ of 1.37 μg/mL [107]. Naturally occurring peptides were the focus of this study, since preclinical models have reported the usefulness of antioxidant peptides in improving cancer therapies. In addition, Omar et al. proved the synergistic effect between methanolic bee-pollen extract of Malaysian stingless bee *L. terminata* and cisplatin [108]. This opens the possibility to apply bee pollen to potentiate the effect of chemotherapy drugs and, simultaneously, reduce the required dose of the drugs.

Similar to honey, the chemoprevention effects are thought to be mainly related to phenolic acids and flavonoids largely present in bee pollen [106,109,110]. These compounds are associated with the stimulation of apoptosis and secretion of tumor necrosis factor-alpha (TNF-α) and the inactivation of oxygen reactive species (ROS), overall inhibiting cell proliferation [1,102,111]. Particularly, the steroid fraction of chloroform extract from bee pollen of *Brassica campestris* L. was shown to induce cytotoxicity, specifically in prostate cancer cells, by triggering apoptosis by activating caspases and downregulation of Bcl-2 expression [112]. In another study, breast cancer cells treated with bee pollen presented early apoptosis features, in contrast to late apoptosis seen in cells treated with propolis [102]. Lastly, although high antiproliferative values and morphological changes related to apoptosis were observed in cells exposed to pollen extract, Mărgăoan et al. found low apoptotic index values for a methanol–water extracted bee pollen collected from *Filipendula ulmaria* [113].

Based on their environmentally friendly properties and chemical composition, bee pollens are also regarded as a promising source for the green synthesis of nanoparticles [110]. Al-Yousef et al. prepared silver nanoparticles using an aqueous extract of bee pollen from a commercial variety of Saudi Arabia as a bioreductant and demonstrated an antiproliferative effect against cancer-cell lines [114].

Overall, the antitumor activity of bee pollen has been mostly assessed using in vitro assays. However, there is insufficient and conflicting evidence to determine the efficacy of bee pollen on cancer [115]. Further in vitro and in vivo studies must be conducted in order to clarify the action mechanisms related to the anticarcinogenic activity of bee pollen.

**Figure 8:**
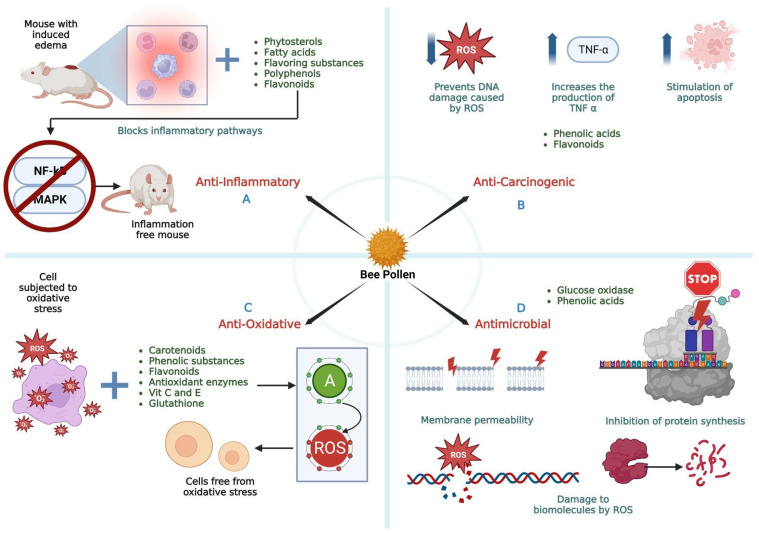
Biological properties of bee pollen. (**A**) Anti-inflammatory activity; (**B**) Anti-carcinogenic activity; (**C**) Anti-oxidative activity; (**D**) Antimicrobial activity. Brown color: components of bee pollen. Blue color: effects of bee pollen. ↑: activation or increase, ↓: decrease or inhibition. Figure created with BioRender.com (accessed on 17 October 2023).

Figure 8 depicts a summary of the principal bee pollen biological activities described in the text.

## 7. Clinical Trials

Clinical trials have assessed the effects of bee pollen consumption for its potential in supporting the immune system and improving prostate illnesses and cancer symptoms. Bee pollen has demonstrated its efficacy in alleviating inflammation in the prostate gland and providing relief to patients with non-bacterial prostate inflammation, benign prostatic hyperplasia, and early-stage prostate cancer [116]. Quercetin, a flavonoid in bee pollen, was suggested to lower oxidative stress and reduce inflammation [117,118]. Clinical studies have further substantiated the hypolipidemic properties of pollen. Also, it may protect the cardiovascular system by lowering lipid and cholesterol levels by 20 to 30%, lowering platelet aggregation, and stabilizing visual acuity in patients with atherosclerotic disease [68]. Initial investigations indicated the potential utility of bee pollen in alleviating symptoms among multiple sclerosis patients, though the quality of supporting evidence was deemed low [119]. Moreover, bee pollen has been applied in cancer clinical trials as a complement to chemotherapeutic treatment and is included in several patents for cancer prevention [116], based on the expected stimulation of the immune system and the protective effect of bee-pollen extracts against chemotherapeutic drug-induced toxicity [99,111]. It is worth noting that the use of bee pollen is not without risks, with potential adverse effects encompassing hepatitis and allergic or hypersensitivity reactions [120].

These clinical trials enhance our comprehension of the potential contributions of bee pollen to health and well-being. Although some of these studies have shown favorable results, research in this domain remains ongoing, and more rigorous investigations are necessary to establish conclusive findings.

## 8. Functional Supplements and Human Health Benefits

Bee pollen has been mainly employed as a food supplement to enhance nutritional value and aid with treating several health conditions, as described in Table 3. Additionally, it has been directly incorporated into some products, including yogurt [121] and bread [122], to improve features not limited only to nutrition, but also including antioxidant qualities, taste, texture, odor, and cohesion. Currently, food supplements of bee pollen rely on blends of pollen from different geographical locations to ensure that the wide variability of the pollen properties becomes manageable, so they offer a more balanced nutritional profile [123]. Table 3 provides a non-exhaustive list of examples of the benefits of pollen consumption for human health, and it aims to highlight the bee pollen potential.

## 9. Side Effects, Allergies, and Food Safety

The increasing emphasis on leading healthy and environmentally conscious lifestyles has driven a growing demand for minimally processed, natural foods enriched with bioactive components such as bee pollen. However, few nations, including Poland, Bulgaria, Switzerland, Brazil, and Argentina, have laws governing bee pollen [12,148]. Currently, there isn’t a document serving as a European standard for beekeeping products. The International Organization for Standardization’s Technical Committee for Food Products established a subcommittee on beekeeping products (ISO/TC34/SC19), which has just started standardizing this natural product. Bee pollen, from diverse origins, is commonly characterized by varying nutritional qualities and food safety issues, due to a lack of regulations [149]. Also, some authors outline potential pollen food-safety issues; for instance, environmental contaminants and harmful chemicals such as pesticides, heavy metals, metalloids, and fungus that produce mycotoxin may contaminate pollen loads [120,149].

Another concern for a particular consumer group is the allergic reactions that may manifest in individuals who are sensitive to pollen. Pollen related-allergy (PRFA) presents symptoms after ingestion and commonly resolves spontaneously within 10–30 min. Most allergic people can tolerate these foods after heating them by boiling, baking, or cooking. However, PRFA should not be overlooked [150]. Bee pollen has 400,000–6,400,000 pollen grains per gram, which might result in severe allergic adverse effects, including anaphylaxis [151].

Focused on the allergens from bee pollen, a study sought to identify and describe the bioactive proteins of pollen from Poland. Purified and concentrated pollen aqueous solutions were examined using the nanoLC-MALDI-TOF/TOF MS analytical technology. The research led to the identification of 197 distinct proteins from green plants (Viridiplantae) and 10 distinct proteins from bees. Potential plant allergens were found in some of them; 65% of the proteins identified in that study were enzymes [152].

Enzymes are sensitizers that can exacerbate allergy symptoms in the respiratory system, such as asthma and rhinitis. Some Proteolytic enzymes are considered allergens because they are released within a few minutes from the pollen grain after being hydrated. The reactions they trigger could be hazardous for some people. Moreover, pollen contains a large amount of proteins, not just enzymes, and their biological actions, functions and interactions within the human body are yet to be studied and better understood [152]. Therefore, it is essential to develop guidelines to standardize bee pollen for human consumption.

The prevalence of documented food allergies has grown in the past few decades, turning them into a matter of public health concern. Alongside more rigorous laws and guidelines for labeling, there is a necessity to increase awareness about food allergies and to advocate for legislative revisions. The absence of standardized labeling regulations and the variation in how allergen information is conveyed can confuse travelers and consumers worldwide [153].

Another important relevant aspect to consider is the consequences of climate change’s influence on human health and its effect on the sources of airborne allergens like pollen and fungal spores. Rising air temperatures and the escalation of atmospheric CO_2_ levels can affect the timing, creation, concentration, allergenic properties, and global spread of airborne allergens such as pollen. This, in turn, will lead to repercussions on allergic respiratory conditions such as allergic asthma and rhinitis [154].

## 10. Conclusions and Future Perspectives

Bee pollen emerges as a remarkably versatile natural compound with a complex composition of diverse bioactive compounds. It is collected by bees for its abundant reserves of proteins, nutrients, and bioactive substances. Furthermore, bee pollen unfolds a spectrum of biological activities, encompassing antioxidant, anti-inflammatory, antimicrobial, antiviral, and anticancer properties. Also, bee pollen includes proteins, carbohydrates, lipids, vitamins, minerals, phenolic compounds, flavonoids, and other bioactive substances, exhibiting varying concentrations and types across different samples. It is crucial to emphasize that the chemical makeup of bee pollen is a dynamic interplay determined by factors such as plant species, geographical location, soil quality, and collection methodology.

Bee pollen’s anti-inflammatory potential is promising, evidenced by its ability to reduce gene expression linked to inflammation and block NF-kB and MAPK signaling pathways. Its substantial antioxidant activity, attributed to a high polyphenolic content, neutralizes free radicals, safeguarding cells from oxidative harm. Regarding antimicrobial prowess, bee pollen demonstrates inhibitory effects against diverse pathogenic bacteria and fungi, suggesting the potential to curb viral replications.

Moreover, research into its anticarcinogenic activity reveals antiproliferative effects on various cancer cell lines, positioning it as a prospective complementary cancer treatment. However, a deeper understanding of the mechanisms underpinning its anticancer potential requires further exploration.

Additionally, bee pollen extracts exhibit a wide range of health benefits, including significant reduction in cholesterol levels, prevention of metabolic syndrome, obesity, atherosclerosis, hyperglycemia, hyperuricemia, myocardial dysfunction, and fluoride toxicity, as well as positive effects on malnourishment, bone metabolism, ovary health, immune stimulation, allergy prevention, cognitive function, and skin conditions, making them a promising and versatile therapeutic agent.

Bee pollen holds immense promise as a functional supplement for human health, owing to its diverse bioactive compounds and therapeutic potential. Continued studies are imperative to unveil its full range of applications and to establish standardized extraction methods for optimal utilization. Throughout history, pollen and bee products have been esteemed for their nutritional and therapeutic properties. Pollen, crucial in pollination and a high-energy food source for bees, bestows numerous nutritional advantages and bioactive compounds. As such, the impact of pollen’s biological activity is significant and currently relevant in food security and health applications.

Based on the findings presented in this review, it is imperative to recognize the inherent complexity of bee pollen’s composition, shaped by variables such as plant species, geographical location, and collection methods. This diversity introduces a level of variability that may impact the generalizability of these results. While our study provides valuable insights into the diverse bioactivities of bee pollen, further research is needed to delve into the underlying mechanisms of bees’ pollen action. Looking ahead, it is crucial for future investigations to address these limitations and this variability to advance our understanding of bee pollen’s biological properties.

Future research on bee pollen should prioritize several key areas to advance our understanding of its biological properties. Establishing standardized extraction methods is crucial to ensure consistency across studies, allowing for accurate comparisons of bee pollen from different sources. Identifying specific active components responsible for its diverse biological activities is essential for targeted applications and therapeutic uses. Further exploration of the anticancer potential demands a detailed investigation into the mechanisms underlying its effects, supported by preclinical and clinical trials.

Safety and toxicity assessments are necessary to establish appropriate dosage guidelines, especially in specific populations or higher doses. Developing standardized products with consistent quality is vital for reliable research outcomes and commercial production. Exploring novel applications in agriculture, food security, and medicine, and investigating synergistic effects with other natural products, will broaden the scope of bee pollen’s potential benefits. In a world grappling with diverse health and environmental challenges, by focusing on these areas, future research endeavors can contribute significantly to maximizing the utility of bee pollen in diverse fields, ultimately enhancing human health and well-being.

## Figures and Tables

**Table 1:** Nutritional content of bee pollen.

Component	Content	Ref
Proteins	2–60%	[6]
	10–40%	[13]
	14–30%	[14]
	22.7%	[15]
Carbohydrates	4–69%	[6]
	15–60%	[13]
	40–85%	[14]
	30.8%	[15]
Lipids	1–20%	[13]
	1–10%	[14]
	4–7%	[6]
	5.1%	[15]
Amino acids	10.4%	[15]
Phenolic compound	1.6%	[15]
Fiber	0.3–20%	[6]

**Table 2:** Nutrient content measurement mechanisms.

Component	Measuring Component	Estimation Method	Ref
Proteins	Nitrogen content	Kjeldahl digestion	[8]
		Dumas combustion	
	Basic amino acid residues content	Bradford colorimetric assay	
	Peptide bonds	Lowry colorimetric assay	
		Bicinchoninic acid (BCA) colorimetric assay	
Carbohydrates	Soluble sugars, starch, and fructosan	Phenol-sulfuric acid colorimetric assay	[8]
Lipids	Lipids content	Folch gravimetric assay	[8]
		Bligh and Dyer gravimetric assay	
		Loveridge gravimetric assay	
		Vanillin colorimetric assay	
Phenolic compound	Phenolic profiles	High-performance liquid chromatography (HPLC)	[20]
Fiber	Soluble and insoluble dietary fiber	Enzymatic gravimetric technique	[21]

**Table 3:** Examples of the benefits of pollen consumption for human health.

Potential Usage	Desired Effect	Results *	Bee Pollen/Extract Type	Ref.
Hypercholesterolemia	Reduction of cholesterol levels	- Reduction of up to 35% of total cholesterol in mice treated with 1 g pollen extract/kg BM.	polyphenol-rich bee pollen ethanol extract	[124]
		- Reduction of triglycerides and cholesterol blood levels in Broiler chickens.	basal diet supplemented with bee-pollen	[125]
		- Reduction of blood cholesterol in patients of the main group of ~0.83 mmol/L. - Reduction of blood triglycerides level in 50% of patients.	aqueous suspension	[126]
Metabolic syndrome	Prevention of metabolic syndrome	- Increase in glutathione S-transferase (GST) and catalase (CAT) activities and decreased the malondialdehyde (MDA) level in mice liver. - Benefited the gut microbiota.	yeast-fermented wall-broken bee pollen	[127]
Obesity	Body weight loss, reduction of lipid accumulation in serum and liver	- Reduction of body weight gains up to 19.37%	ethanol extract	[128]
Atherosclerosis	Reduction or prevention of chronic inflammation in the aorta and medium-sized arteries	- Prevention of atherosclerosis - occurrence (the dose of 1 g/kg BM) in mice.	ethanol extract	[129]
Hyperglycemia	Reduction of blood glucose levels	- Inhibition of α-glucosidase - IC50 0.60 mg/mL compared to IC50 11.3 mg/mL of the control in vitro.	water extracts	[130]
		- Reduction of fasting blood glucose (FBG) levels in male Wistar rats.	bee pollen	[131]
		- Reduction of fasting blood glucose (FBG) level of 5 mmol/L of blood sugar in rats.	bee pollen	[132]
		- Reduction of fasting blood glucose (FBG) of ~61.34 mg/ dL in rats.	bee pollen suspension	[133]
		- Reduction in blood glucose on average by 22.3% compared with the values before the treatment in diabetic patients.	aqueous suspension	[126]
Diabetic testicular-pituitary system dysfunction	Protection against diabetes-induced dysfunction	- Enhancement of the testicular antioxidant defense systems. - Increase of glutathione-S-transferase (GST), glutathione (GSH), superoxide dismutase (SOD), and glutathione peroxidase (GPX) in Wistar rats.	suspension	[133]
Hyperuricemia	Reduction of uric acid levels	- Reduction of up to 43% in serum uric acid. - Inhibition of liver xanthine oxidase activity of up to 34% in mice.	n-butanol extract	[134]
		- Reduction of up to 73% in serum uric acid. - Improvement of renal function. - Inhibition of liver xanthine oxidase activity and expression regulation of the of urate transporter 1 (URAT1), glucose transporter 9 (GLUT9), organic anion transporter 1 (OAT1), organic cation transporter 1 (OCT1), and ATP-binding cassette superfamily gmember 2 (ABCG2) in mice kidney.	ethyl acetate extract	[135]
Myocardial dysfunction and damage	Cardioprotective agent	- Reduction of serum aspartate transaminase, lactate dehydrogenase, and creatine kinase activities. - Increase of myocardial superoxide dismutase, glutathione peroxidase, and catalase activities.	ethanol extract	[136]
Fluorosis	Reduction of the fluoride toxic effects	- Reduction in MDA level and increase in erythrocyte superoxide dismutase (SOD) activity, glutathione (GSH) levels in rat blood and brain. - Reduction in ALP activity, urea, creatinine, sodium, and potassium levels in serum.	bee pollen	[137]
Malnourishment	Source of essential amino acids, fatty acids, and micronutrients	- Significant increase in muscle mass, restoration of protein synthesis rate and mTOR/p70S6kinase/4eBP1 activation, as well as improvement of mitochondrial activity in rats.	bee pollen	[1]
Bone metabolism	Preventive effects on bone loss	- Prevention of calcium levels and alkaline phosphatase activity reduction in diabetic rats.	water extract	[138]
		Increase in calcium content in metaphyseal and diaphyseal tissues. Inhibition of osteoclastic bone resorption in rats.		[139]
Ovary health	Regulation of the ovarian functions	- Reduction of insulin-like growth factor I (IGF-I). - Increase of steroid hormones (progesterone and estradiol). - Increase of expression of markers of apoptosis (Bcl-2, Bax and caspase-3) in rats.	rape seed bee pollen	[140]
		- Reduction of insulin-like growth factor I (IGF-I). - No changes on progesterone levels and proliferation markers and caspase-3 in vitro.	rape seed bee pollen	[141]
	Prevention of polycystic ovary syndrome	- Increase of preantral and antral follicles. - Reduction of cystic follicles. - Reduction of the levels of TNF-α, NO, as well as the expressions of Ki67. - Increase of apoptosis in the groups treated with bee pollen.	suspension	[142]
Immunostimulant	- Improvement of hematological parameters and antioxidant enzymes. - Reduction of inflammatory cytokines.	- Improvement of hematopoietic function, antioxidant parameters, and serum levels of hematopoietic stimulating-cytokines. - Reduction of the expression of apoptotic genes in rats.	water extract	[143]
Anti-allergic agent	Prevention of allergy	- Reduction of the cutaneous mast cell activation elicited by IgE and specific antigens. - Reduction of in vitro mast cell degranulation and tumor necrosis factor-α production.	phenolic extract	[144]
		- Inhibition of both IgG1and IgE ovalbumin (OVA)-specific production in mice. - Partial protection on the OVA-induce anaphylactic shock reaction.	phenolic extract	[145]
Cognitive dysfunction	Improvement of cognitive impairment	- Increase of the conversion of pro-brain-derived neurotrophic factor (BDNF) to mature BDNF by tissue plasminogen activator.	ethanol extract	[146]
Skin conditions	Against abnormal melanogenesis, hyperpigmentation	- Inhibition of intracellular anti-tyrosinase (TYR) activity, reduction of melanin content, and increase of glutathione synthesis. - Reduction of mRNA expression of TYR, TYR-related protein (TRP)-1 and TRP-2, and inhibition of cyclic adenosine monophosphate (cAMP) in vitro.	phenolic extracts	[78,147]

* Compared to their respective control groups.

## Data Availability

No new data were created or analyzed in this study. Data sharing is not applicable to this article.
